# Supplementary material for: Genotoxic antibody-drug conjugates combined with Bcl-xL inhibitors enhance therapeutic efficacy in metastatic castration-resistant prostate cancer
Source: bioRxiv. 2025 Sep 11:2025.09.05.674562. Preprint. [Version 1] doi: 10.1101/2025.09.05.674562 (PMC12439934; doi:10.1101/2025.09.05.674562)
Supplement: Supplement 4 [file media-4.pdf]

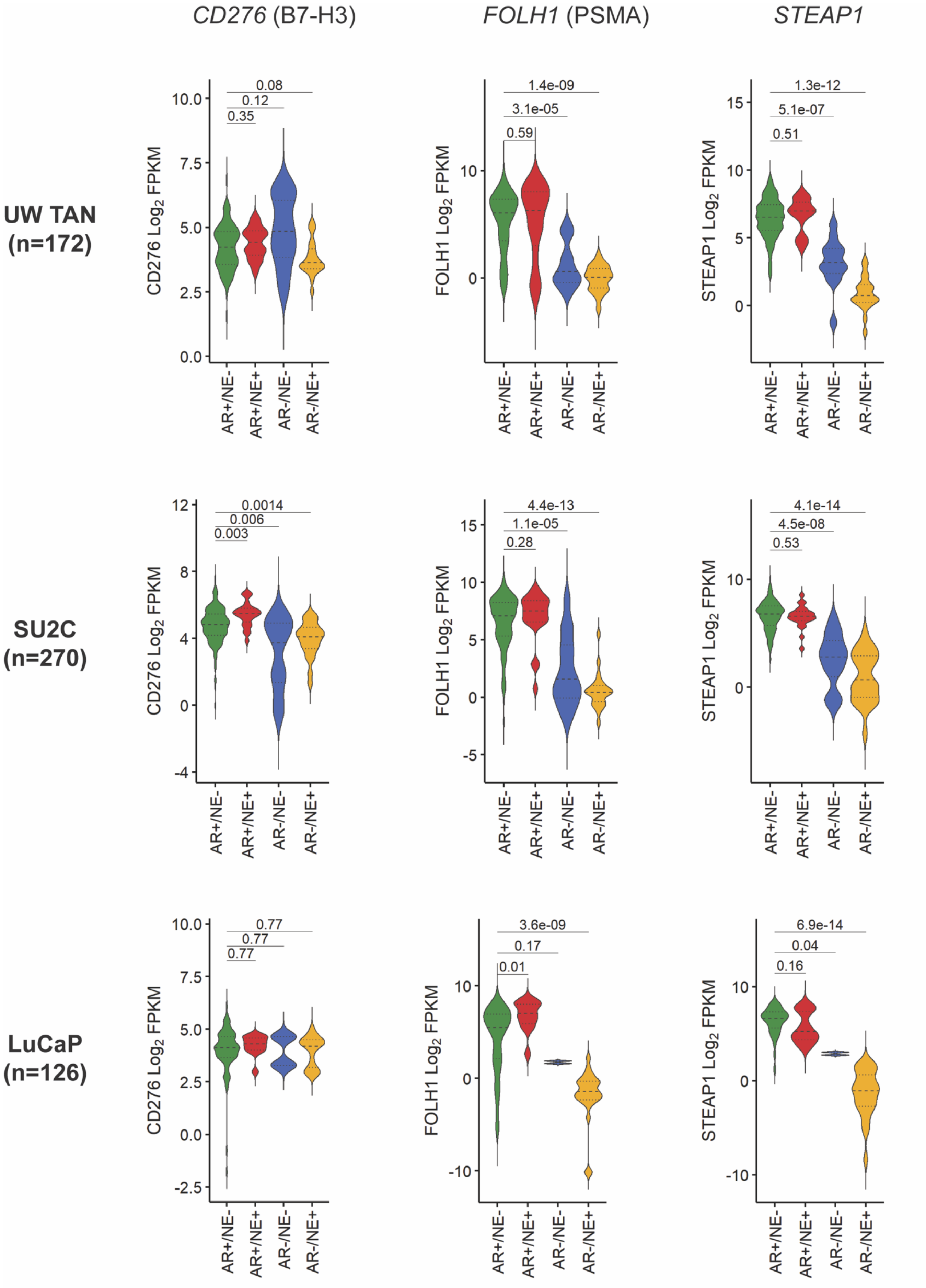
CD276 (B7-H3)
FOLH1 (PSMA)
STEAP1
UW TAN (n=172)
SU2C (n=270)
LuCaP (n=126)
CD276 Log2 FPKM
FOLH1 Log2 FPKM
STEAP1 Log2 FPKM
0.08
0.12
0.35
1.4e-09
3.1e-05
0.59
1.3e-12
5.1e-07
0.51
0.0014
0.006
0.003
4.4e-13
1.1e-05
0.28
4.1e-14
4.5e-08
0.53
0.77
0.77
0.77
3.6e-09
0.17
0.01
6.9e-14
0.04
0.16
AR+/NE-
AR+/NE+
AR-/NE-
AR-/NE+

Figure S1. *CD276* (B7-H3), *FOLH1* (PSMA), and *STEAP1* expression across mCRPC molecular subtypes.

Violin plots show *CD276*, *FOLH1*, and *STEAP1* transcript levels in AR+/NE-(green), AR+/NE+(red), AR-/NE-(blue), and AR-/NE+(yellow) tumors from UW TAN, SU2C, and LuCaP cohorts. Results are expressed as log2 fragments per kilobase of transcript per million mapped reads (FPKM). The groups were compared using two-sided Wilcoxon rank tests with Benjamini-Hochberg multiple-testing correction.

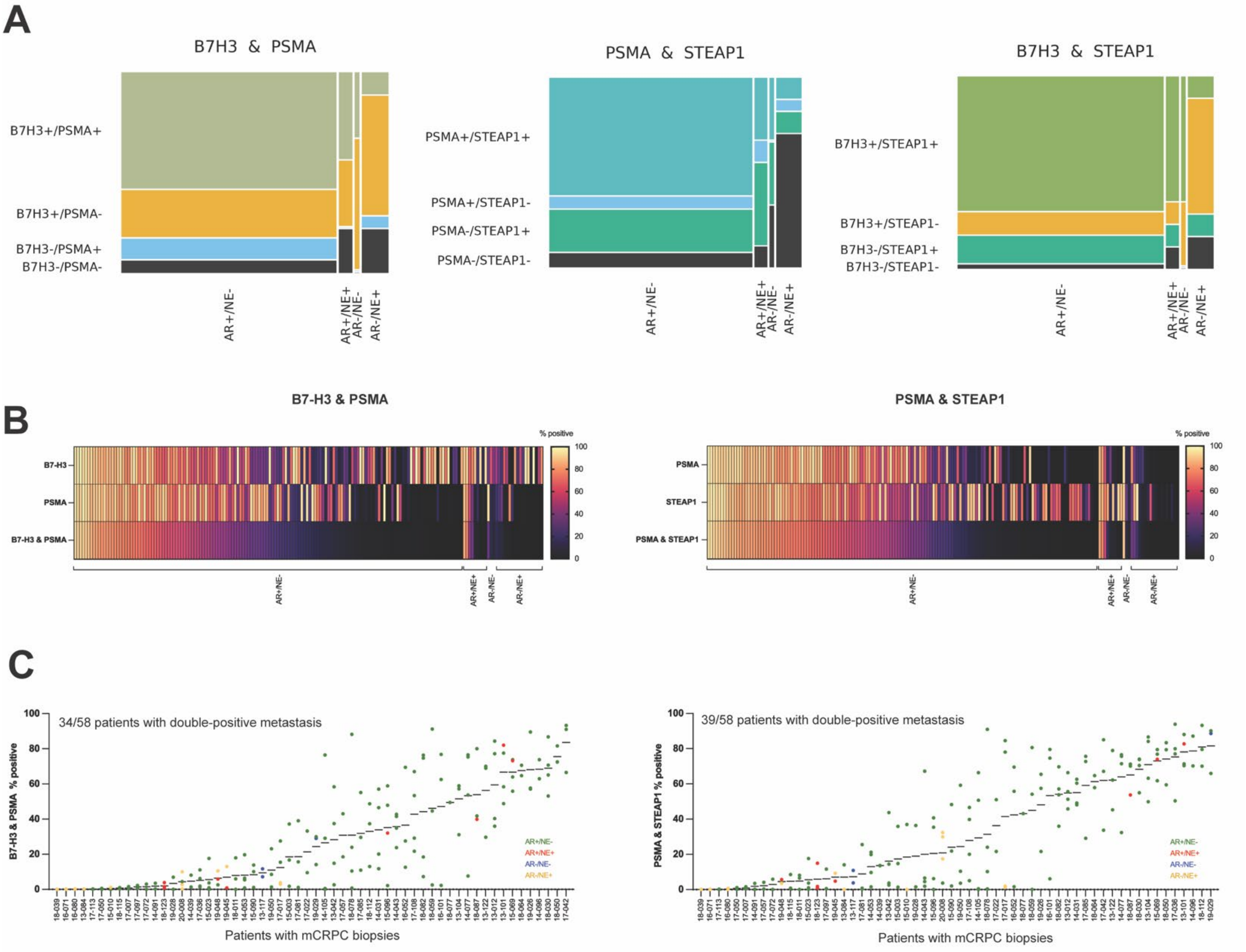

Figure S2. Co-expression of B7-H3 and PSMA / PSMA and STEAP1 / B7-H3 and STEAP1 in mCRPC tumors and patients.

(A) Mosaic plot showing mCRPC subtypes scaled to their relative proportions versus marker status pairs scaled to their relative proportions within each subtype. Double positivity is defined as ≥20% positive cells. (B) Heatmaps showing percents of cells staining positively for B7-H3 and PSMA (left) or PSMA and STEAP1 (right) in each individual mCRPC tumor (columns, n=176). (B). Distribution of B7-H3 and PSMA (left) or PSMA and STEAP1 (right) double-positive cells in 176 metastatic tumors within and between 58 patients from UW TAN cohort. Each dot represents a tumor sample; the color codes indicate the molecular subtype – AR+/NE- (green), AR+/NE+ (red), AR-/NE- (blue), and AR-/NE+ (yellow).

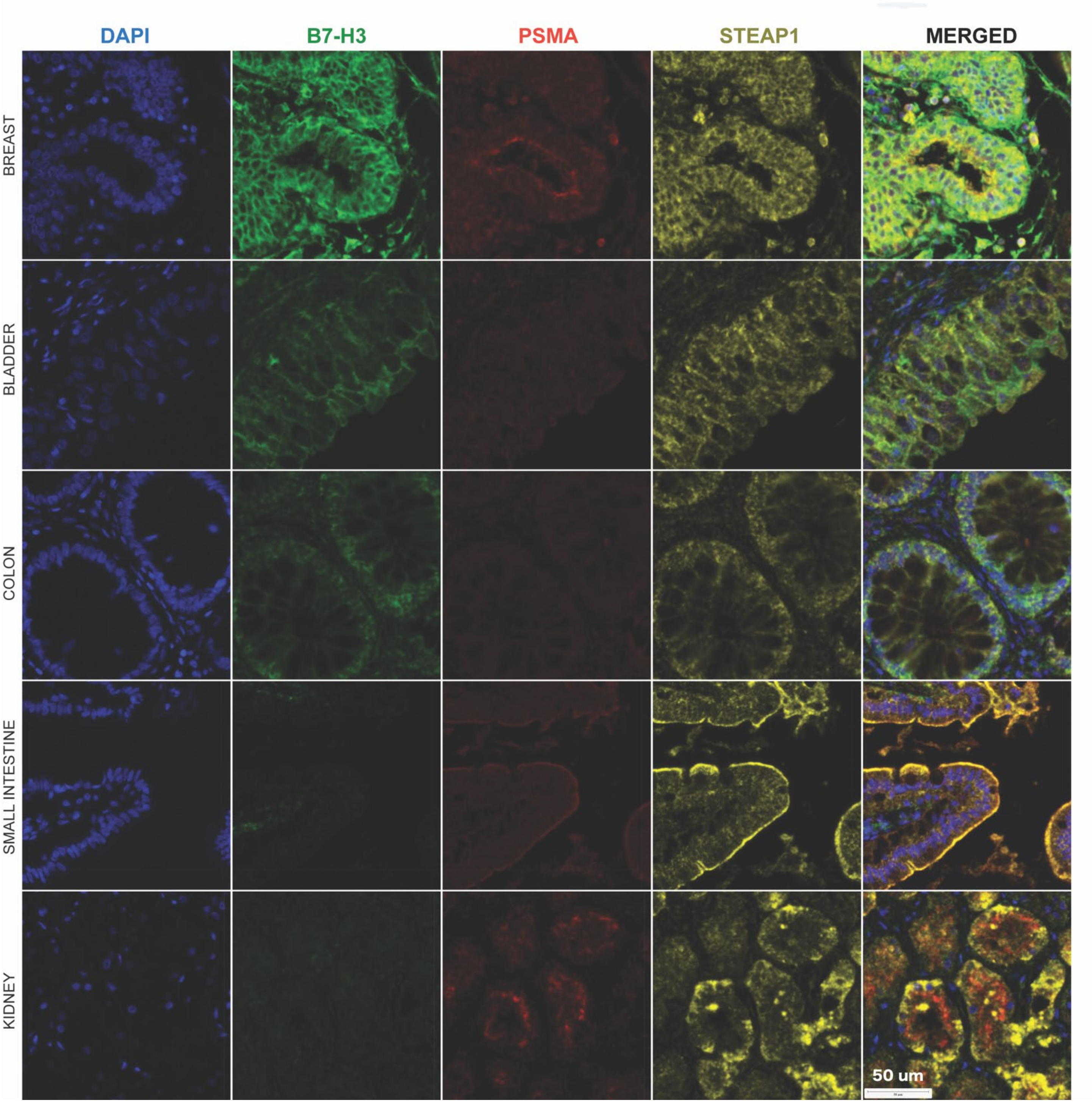

Figure S3. Normal tissues co-expressing B7-H3, PSMA, and STEAP1.

Representative TMA images of human breast, bladder, kidney, small and large intestine tissues (FDA999 L206) with membranous B7-H3, PSMA, STEAP1, and nuclear DAPI staining.

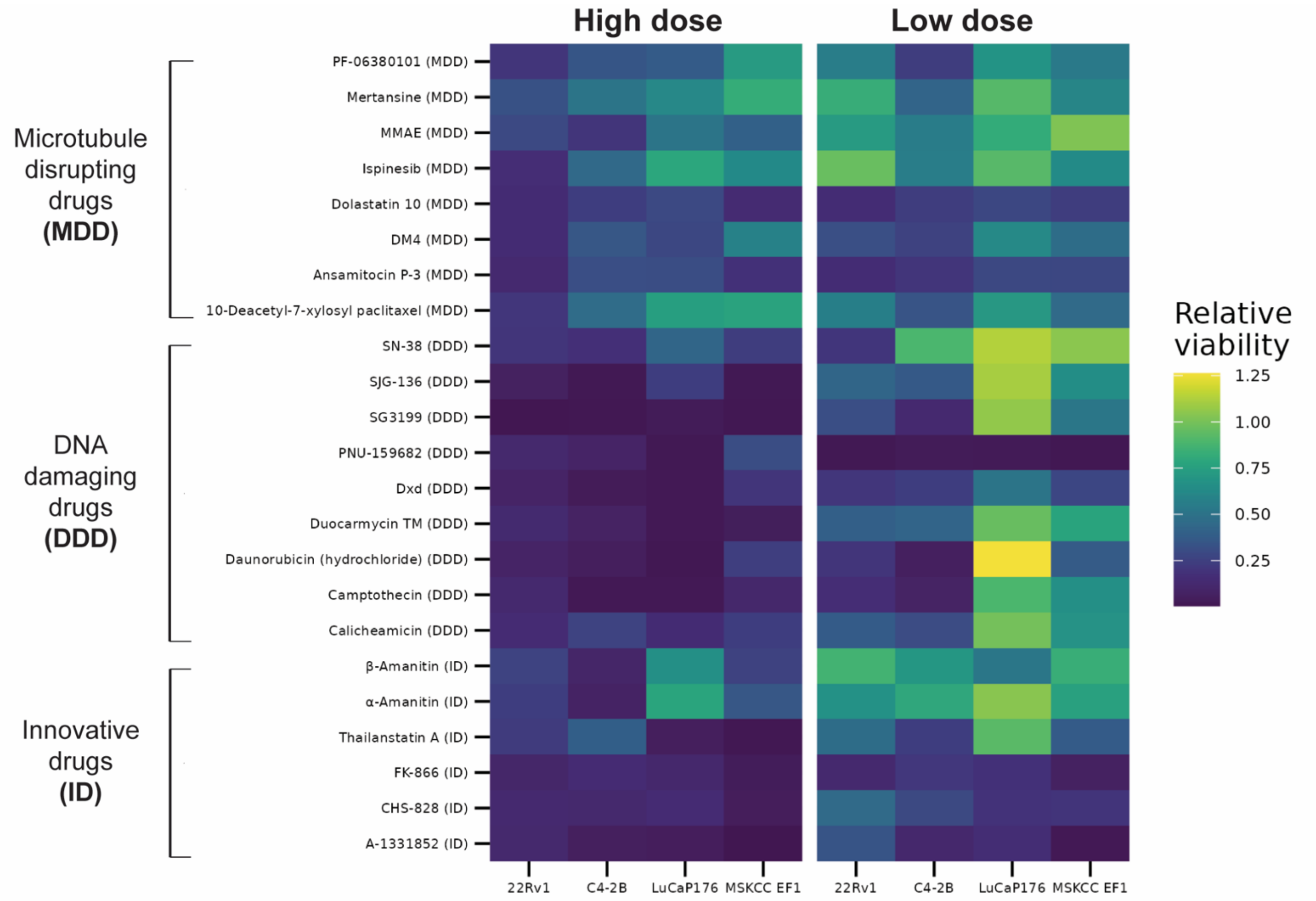

Line	Group 1	Group 2	n 1	n 2	Low dose Estimate (95% CI)	Low dose P-value	High dose Estimate (95% CI)	High dose P-value
**C4-2B**	MDD	DDD	8	9	0.09 (0.03, 0.17)	0.005	0.13 (-0.10, 0.28)	>0.9
	MDD	ID	8	6	0.03 (-0.08, 0.10)	0.5	0.01 (-0.44, 0.26)	>0.9
	DDD	ID	9	6	-0.07 (-0.14, 0.00)	0.18	-0.10 (-0.46, 0.15)	>0.9
**22Rv1**	MDD	DDD	8	9	0.28 (0.17, 0.41)	<0.001	0.31 (-0.05, 0.55)	0.2
	MDD	ID	8	6	0.23 (0.09, 0.36)	0.02	0.07 (-0.31, 0.43)	0.8
	DDD	ID	9	6	-0.06 (-0.13, 0.03)	0.11	-0.24 (-0.47, 0.03)	0.2
**LuCaP176**	MDD	DDD	8	9	0.36 (0.26, 0.60)	0.003	-0.27 (-0.58, 0.02)	0.18
	MDD	ID	8	6	0.24 (-0.27, 0.57)	0.18	0.13 (-0.24, 0.63)	0.5
	DDD	ID	9	6	-0.10 (-0.64, 0.03)	0.18	0.41 (-0.05, 0.88)	0.2
**MSKCC EF1**	MDD	DDD	8	9	0.41 (0.13, 0.59)	0.02	-0.06 (-0.33, 0.26)	>0.9
	MDD	ID	8	6	0.44 (0.14, 0.72)	0.02	0.22 (-0.30, 0.52)	>0.9
	DDD	ID	9	6	0.02 (-0.12, 0.21)	0.6	0.23 (-0.19, 0.60)	>0.9

Figure S4. Prostate cancer cell lines demonstrate greater response to DNA-damaging drugs (DDD) compared to microtubule-disrupting drugs (MDD).

Heatmap (top) visualizes relative viability by drug and cell line for high and low doses. RLU – relative luminescence units (relative cell viability). Pairwise comparisons of relative cell viability between payload groups (bottom). The groups were compared using Wilcoxon-Mann-Whitney test.

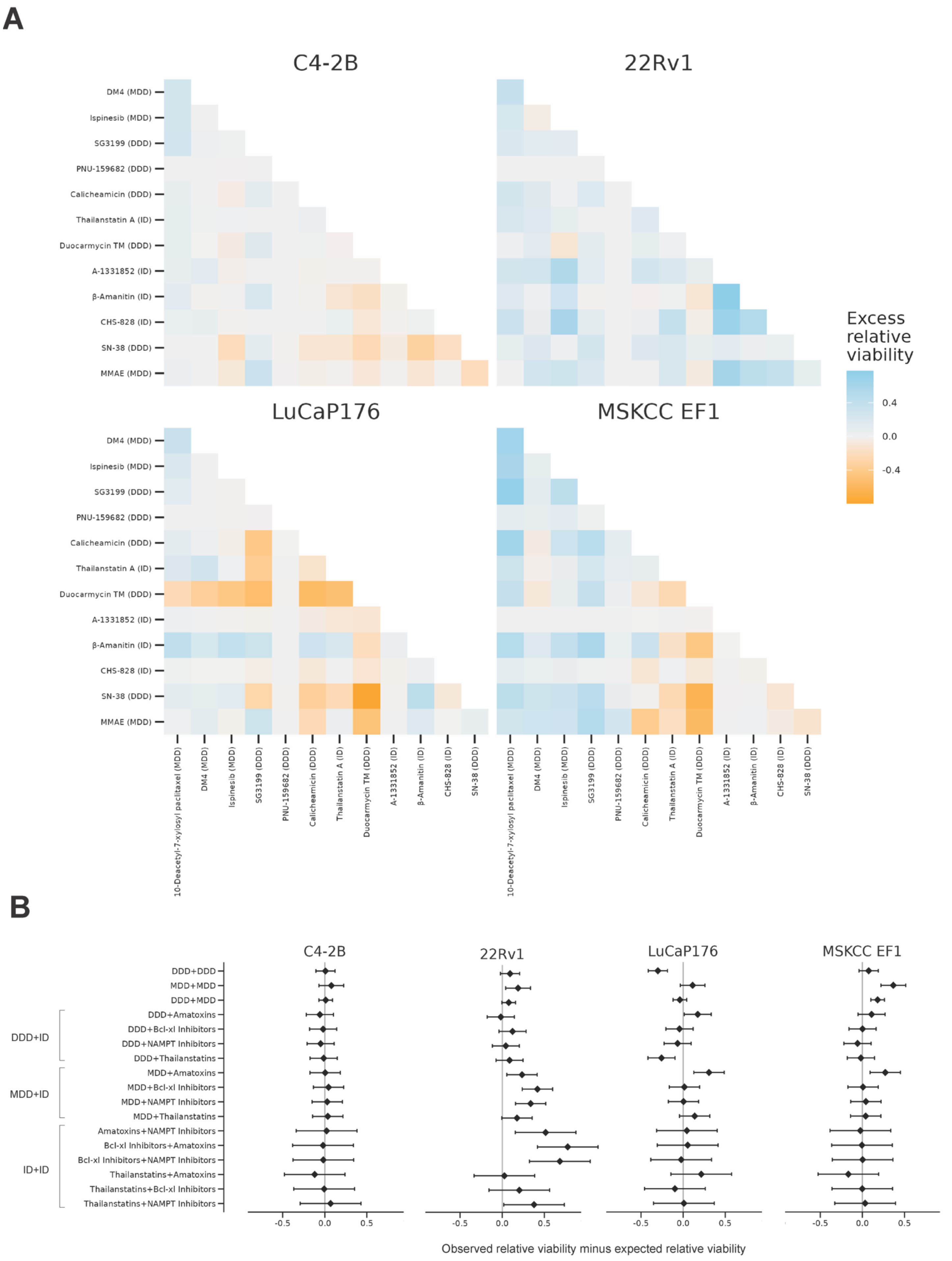
A
C4-2B
22Rv1
LuCaP176
MSKCC EF1
DM4 (MDD)
Ispinesib (MDD)
SG3199 (DDD)
PNU-159682 (DDD)
Calicheamicin (DDD)
Thailanstatin A (ID)
Duocarmycin TM (DDD)
A-1331852 (ID)
β-Amanitin (ID)
CHS-828 (ID)
SN-38 (DDD)
MMAE (MDD)
10-Deacetyl-7-xylosyl paclitaxel (MDD)
Excess relative viability
0.4
0.0
-0.4
B
DDD+DDD
MDD+MDD
DDD+MDD
DDD+Amatoxins
DDD+Bcl-xl Inhibitors
DDD+NAMPT Inhibitors
DDD+Thailanstatins
MDD+Amatoxins
MDD+Bcl-xl Inhibitors
MDD+NAMPT Inhibitors
MDD+Thailanstatins
Amatoxins+NAMPT Inhibitors
Bcl-xl Inhibitors+Amatoxins
Bcl-xl Inhibitors+NAMPT Inhibitors
Thailanstatins+Amatoxins
Thailanstatins+Bcl-xl Inhibitors
Thailanstatins+NAMPT Inhibitors
DDD+ID
MDD+ID
ID+ID
-0.5
0.0
0.5
Observed relative viability minus expected relative viability

Figure S5. Combination payload screening prioritizes candidates for synergy assessment.

(A) Heatmaps showing excess relative viability of C4-2B, 22Rv1, LuCaP 176, and MSKCC EF1 cells exposed to single payloads at low dose and payload combinations. Excess viability was calculated as *observed viability* ($viability_{drug1+drug2}$) - *expected viability* ($viability_{drug1}$ * $viability_{drug2}$). (B). Excess relative viability means for the combinations between payload groups and classes in four cell lines.

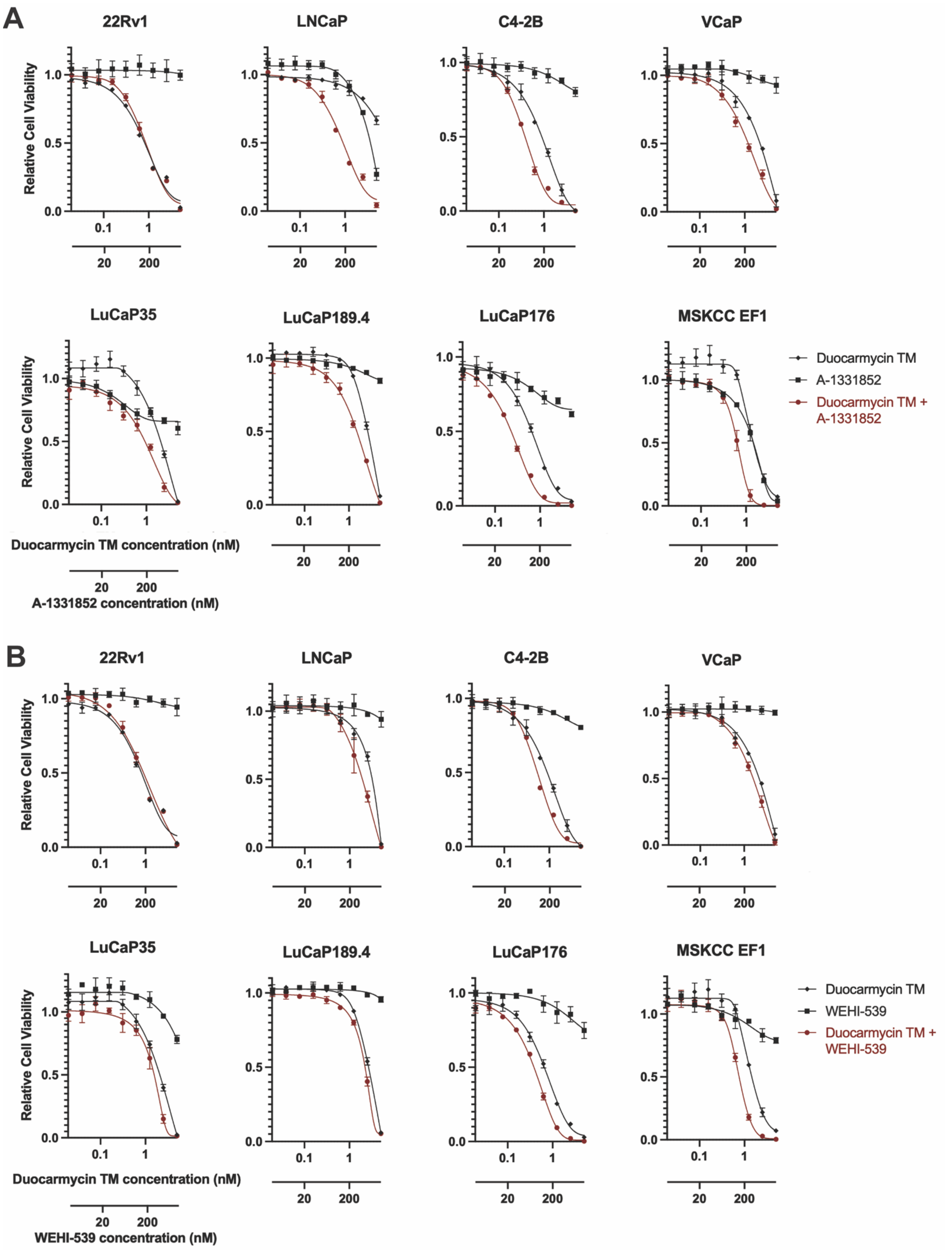
A
22Rv1
LNCaP
C4-2B
VCaP
LuCaP35
LuCaP189.4
LuCaP176
MSKCC EF1
Relative Cell Viability
Duocarmycin TM concentration (nM)
A-1331852 concentration (nM)
Duocarmycin TM
A-1331852
Duocarmycin TM + A-1331852
B
22Rv1
LNCaP
C4-2B
VCaP
LuCaP35
LuCaP189.4
LuCaP176
MSKCC EF1
Relative Cell Viability
Duocarmycin TM concentration (nM)
WEHI-539 concentration (nM)
Duocarmycin TM
WEHI-539
Duocarmycin TM + WEHI-539

Figure S6. Duocarmycin TM and Bcl-xL inhibitors (A-1331852 and WEHI-539) combinations exhibit synergistic cytotoxicity in a panel of prostate cancer cell lines.

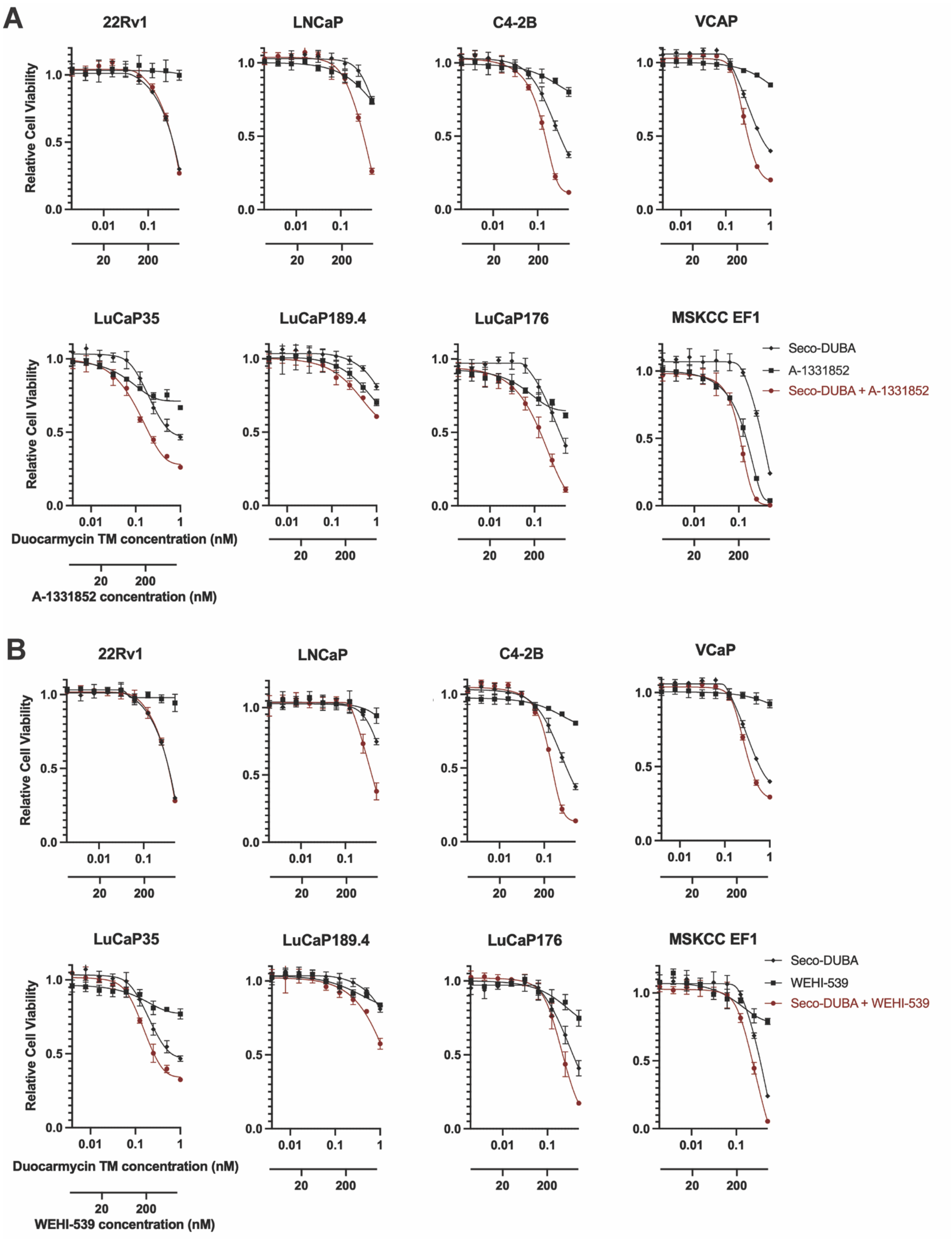
A
22Rv1
LNCaP
C4-2B
VCAP
Relative Cell Viability
1.0
0.5
0.0
0.01
0.1
1
20
200
LuCaP35
LuCaP189.4
LuCaP176
MSKCC EF1
Seco-DUBA
A-1331852
Seco-DUBA + A-1331852
Duocarmycin TM concentration (nM)
A-1331852 concentration (nM)
B
22Rv1
LNCaP
C4-2B
VCaP
Relative Cell Viability
LuCaP35
LuCaP189.4
LuCaP176
MSKCC EF1
Seco-DUBA
WEHI-539
Seco-DUBA + WEHI-539
Duocarmycin TM concentration (nM)
WEHI-539 concentration (nM)

Figure S7. Seco-DUBA and BCL-XL inhibitors (A-1331852 and WEHI-539) combinations exhibit synergistic cytotoxicity in a panel of prostate cancer cell lines.

A

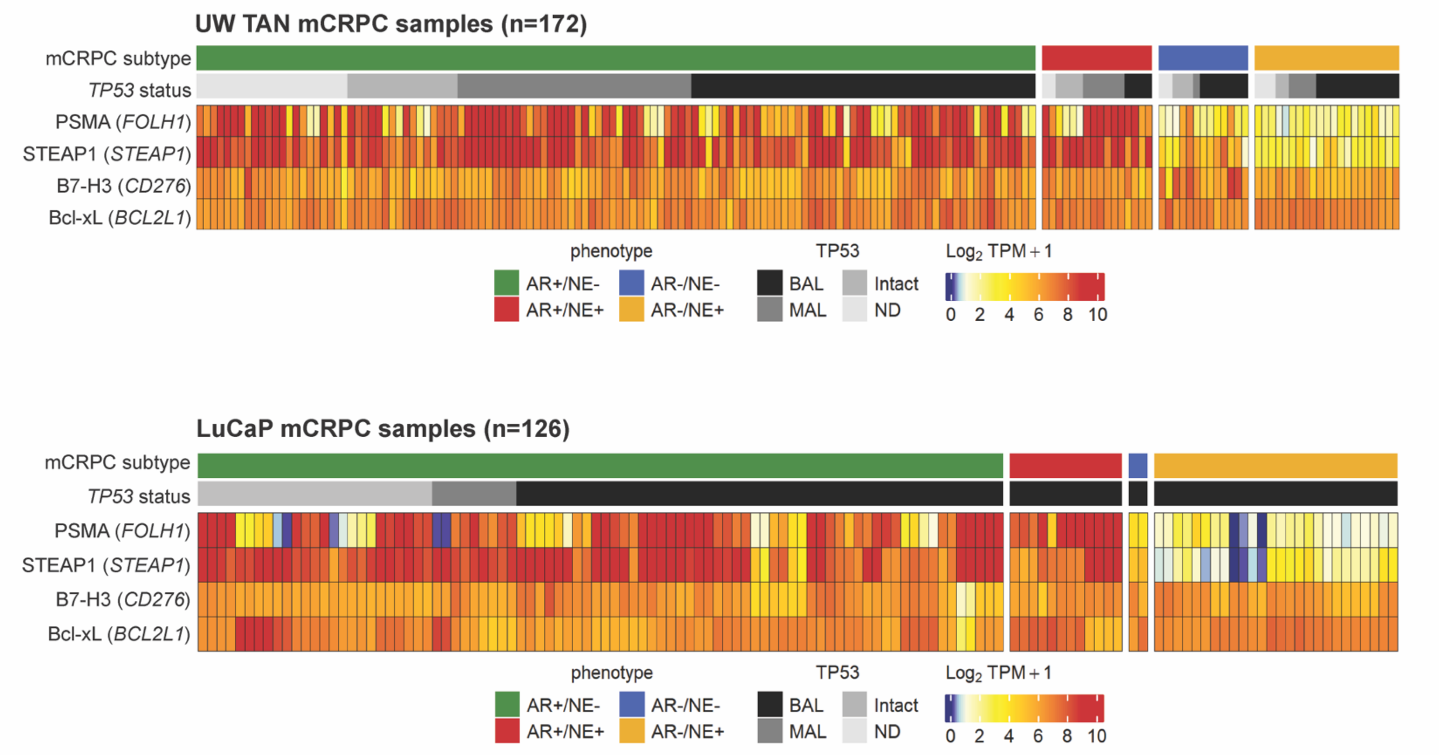

B

Dataset	BAL	Intact	MAL	ND	Total	%BAL
SU2C	95	68	91	16	270	35%
AR+/NE-	71	58	69	12	210	34%
AR+/NE+	10	3	10	0	23	43%
AR-/NE-	3	4	5	2	14	21%
AR-/NE+	11	3	7	2	23	48%
UW TAN	73	25	45	29	172	42%
AR+/NE-	50	16	34	22	122	41%
AR+/NE+	4	4	6	2	16	25%
AR-/NE-	7	3	1	2	13	54%
AR-/NE+	12	2	4	3	21	57%
LuCaP	92	25	9	0	126	73%
AR+/NE-	52	25	9	0	86	60%
AR+/NE+	12	0	0	0	12	100%
AR-/NE-	2	0	0	0	2	100%
AR-/NE+	26	0	0	0	26	100%

Figure S8. *TP53* genomic alterations in mCRPCs expressing B7-H3 (*CD276*), PSMA (*FOLH1*), STEAP1, and Bcl-xL (*BCL2L1*).

(A) Heatmap showing *FOLH1, STEAP1, CD276,* and *BCL2L1* transcript abundance, as well as *TP53* genomic status in UW TAN and LuCaP mCRPC specimens. Transcript levels are shown as Log_2 TPM + 1. BAL – biallelic loss, MAL – monoallelic loss, ND – no data. (B) Fractions of tumors with and without

TP53 genomic alterations in SU2C, UW TAN, and LuCaP cohorts across 4 mCRPC molecular subtypes.

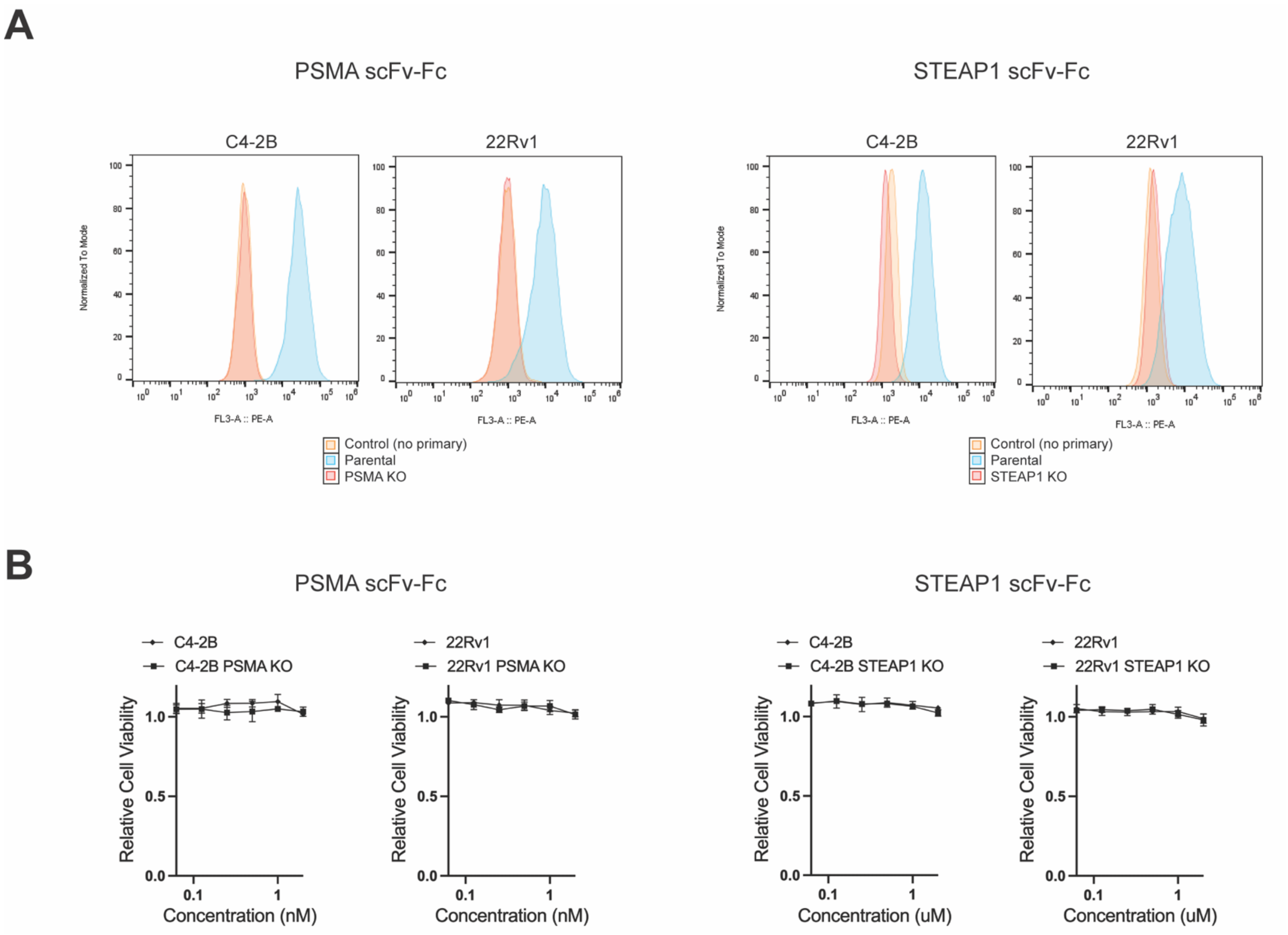

Figure S9. Quality control of PSMA and STEAP1 scFv-Fc antibodies.

(A) PSMA scFv-Fc or STEAP1 scFv-Fc binding to the target antigens determined by flow cytometry in parental and antigen KO C4-2B and 22Rv1 cells. (B) Viability of C4-2B and 22Rv1 cells exposed to naked PSMA scFv-Fc or STEAP1 scFv-Fc.

A

Group * Day	C4-2B			C4-2B *TP53 KO*		
	exp(Beta)	95% CI[1]	p-value	exp(Beta)	95% CI[1]	p-value
B7-H3 – seco-DUBA* Day	0.95	0.94, 0.96	<0.001	0.97	0.96, 0.98	<0.001
A1331852 * Day	0.97	0.96, 0.98	<0.001	0.99	0.98, 1.00	0.065
B7-H3 – seco-DUBA+A1331852 * Day	0.94	0.93, 0.94	<0.001	0.96	0.95, 0.96	<0.001

B

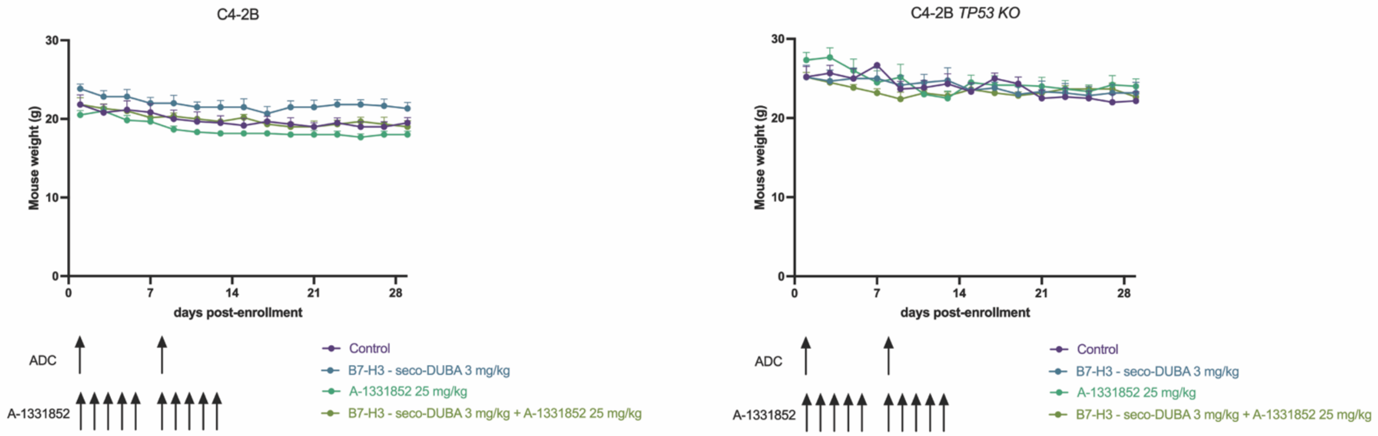

Figure S10. Analysis of volumetric changes in CDX tumors and body weight changes in animals from control and treatment C4-2B and C4-2B *TP53 KO* groups.

(A) Statistical analysis for the estimated tumor volume growth rate with tests relative to the control. CI[1] – confidence interval. (B) Average body weights per group throughout the experiment. Error bars represent standard error of the mean.
